# The relationship between body mass index and types of peer bullying in primary school students: rising risk in cyberbullying

**DOI:** 10.3389/fpubh.2026.1917762

**Published:** 2026-09-09

**Authors:** Yasemin Büyükşahin, Esma Nur Biçim

**Affiliations:** Department of Primary Education, Faculty of Education, Bartin University, Bartin, Türkiye

**Keywords:** body mass index, cyberbullying, obesity, peer bullying, primary school students

## Abstract

**Introduction:**

Childhood obesity and peer bullying are significant psychosocial challenges in primary education. This study investigated the predictive effect of Body Mass Index (BMI) categories on various types of peer bullying victimization among primary school students.

**Methods:**

Using a correlational screening model, data were collected from 579 students (Grades 1–4) in Bartin, Türkiye, during the 2025–2026 academic year. Instruments included a six-item peer bullying questionnaire and standardized physical measurements. BMI was categorized according to WHO (2007) references. Overweight and obese groups were merged for comparison against the normal-weight group. Statistical analyses included chi-square tests, binary logistic regression, and Pearson/Spearman correlations.

**Results:**

While BMI category did not significantly predict general victimization, cyberbullying emerged as a consistent exception. In the unadjusted logistic regression, overweight/obese students were 1.83 times more likely to experience cyberbullying [OR = 1.83, 95% CI (1.008, 3.328), *p* = 0.047]; a permutation test confirmed this was unlikely to reflect chance (*p*_perm_ = 0.032). When adjusted for gender and grade, this association showed borderline significance (OR = 1.77, *p* = 0.064). Correlation analyses confirmed a positive relationship between continuous BMI and cyberbullying (ρ = 0.092, *p* = 0.027).

**Discussion:**

Higher BMI is more closely associated with cyber victimization than physical or social bullying. Overweight children may face increased digital risks due to social withdrawal. Prevention programs should address the “invisibility” of cyberbullying and encourage the broader school community—including teachers—to avoid stigmatizing language and behaviors.

## Introduction

Childhood obesity is considered one of the fastest-growing public health problems worldwide. According to World Health Organization data, the prevalence of obesity among children and adolescents aged 5–19 has increased dramatically over the past four decades, transforming obesity from a purely biomedical issue into a complex phenomenon with social and psychological dimensions. The etiology of childhood obesity rests on the complex interplay of biological predispositions, environmental factors, and maternal influences (1), placing obese children in a vulnerable position both physically and psychosocially. Studies show that obese children at school are more often described as socially withdrawn and less attractive, more frequently perceived as those who are teased or involved in fights, and less often identified as best friends by classmates (2). This pattern suggests that body weight can profoundly shape a child's social world.

Peer bullying is defined as a set of unwanted behaviors occurring repeatedly among individuals characterized by a power imbalance, manifesting in physical (hitting, pushing), verbal (name-calling, teasing), emotional (exclusion, gossip), or digital forms (3, 4). This problem is widely observed among school-age children worldwide and has adverse effects on victims' academic achievement and psychological well-being (5). Research in Türkiye confirms that physical bullying is particularly prevalent among male students (5, 6), and bullying victimization has been associated with psychopathologies such as alcohol use and eating disorders (7).

For children of the alpha generation growing up immersed in digital technology, bullying is no longer confined to school corridors. Cyberbullying operates as a bidirectional mechanism: the bullied child withdraws from social environments and increasingly spends time on digital platforms, which in turn renders the child more vulnerable to online victimization (8). Cyberbullying victimization has been reported to be as devastating to mental health as traditional school bullying (9), and its invisibility and omnipresence enable a continuous cycle of victimization (10).

Research examining physical differences and peer bullying has revealed that overweight or obese children face a higher risk of bullying than normal-weight peers (11, 12). Physical attributes such as BMI are thought to increase bullying risk, and negative attitudes toward obese or underweight children have been observed (13). Body size can directly influence acceptance or rejection in peer relationships, and children with different physical characteristics may be frequently excluded (5). Obesity-related body image differences may trigger exclusion, labeling, and self-esteem and self-worth problems (13). Nevertheless, studies systematically examining the relationship between BMI and different types of bullying—especially in primary school-age children in Türkiye—remain quite limited.

Existing bullying research in Türkiye has largely focused on gender, grade level, and socioeconomic variables; the potential role of body weight on peer bullying has not been adequately addressed. Yet, understanding how peers perceive a child's body weight and how this shapes bullying behaviors could provide critical information for prevention and intervention programs. Given that teachers' stigmatizing remarks about obesity can trigger peer bullying (14), examining these dynamics holistically is of great importance. Children who face discrimination based on physical appearance are not only bullying victims but may eventually shift to the perpetrator role (15).

Drawing from this need, the present study aims to examine the relationship between BMI category and victimization across six types of peer bullying—physical bullying, unauthorized taking of belongings, unwanted name-calling, upsetting pranks, mockery, and cyberbullying—among Grade 1–4 students (*N* = 579) in a public primary school in Bartin, Türkiye. The central research question is: *Does BMI category (Normal / Overweight-Obese) significantly predict the likelihood of victimization across types of peer bullying in primary school students?* Potential confounding effects of gender and grade level were also controlled.

## Method

### Research design

This study employed a quantitative relational screening model, which aims to describe the relationship between two or more variables in their existing state without any intervention (16). BMI category served as the independent variable and peer bullying victimization status as the dependent variable. This study was reported in accordance with the STROBE guidelines for observational studies.

### Participants

The study group consisted of Grade 1–4 students enrolled at a public primary school in Bartin, Türkiye, during the 2025–2026 academic year. A census method was adopted, targeting all primary school students at the school. A total of *N* = 579 students participated; all students had complete height and weight measurements, and no cases were excluded due to missing physical data. As a census-based study targeting all eligible students at the school, no a priori sample size calculation was conducted; the achieved sample of *N* = 579 was considered adequate for the planned logistic regression analyses given the observed event rates. Participant distribution by grade and sex is presented in Table 1.

**Table 1:** Participant distribution by grade level and sex.

Grade level	Girls *n*	Girls %	Boys *n*	Boys %	Total *N*
Grade 1 (Age 7)	58	43.9%	74	56.1%	132
Grade 2 (Age 8)	74	51.7%	69	48.3%	143
Grade 3 (Age 9)	69	51.1%	66	48.9%	135
Grade 4 (Age 10)	78	46.2%	91	53.8%	169
**Total**	**279**	**48.2%**	**300**	**51.8%**	**579**

**Figure 1:**
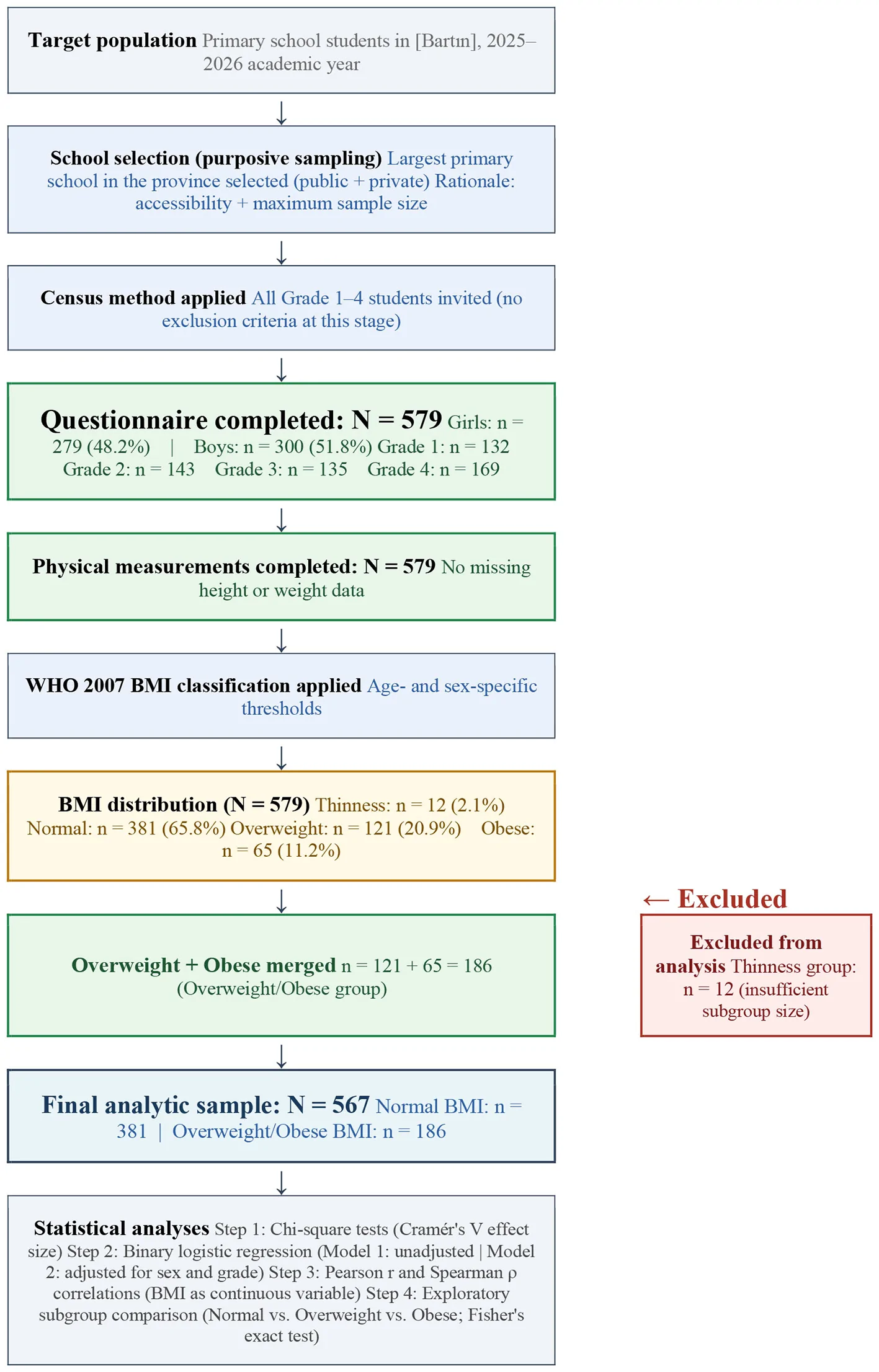
STROBE flow diagram: participant selection and BMI classification process. BMI, Body Mass Index. WHO, World Health Organization. Purposive sampling was used at the school-selection stage; the largest primary school across all public and private schools in the province was chosen to maximize sample size. A census approach was then applied to all enrolled Grade 1–4 students. No students were excluded due to missing physical measurement data.

Age assignment is based on grade level (Grade 1 = 7 years, Grade 2 = 8 years, Grade 3 = 9 years, Grade 4 = 10 years). Participant flow and sampling decisions are detailed in Figure 1 (STROBE flow diagram).

### Measures

***Peer Bullying Questionnair ***. A researcher-developed questionnaire measured students‘ exposure to six types of bullying: (1) physical bullying (hitting, pushing, etc.), (2) unauthorized taking of belongings, (3) unwanted name-calling, (4) upsetting pranks, (5) mockery, and (6) cyberbullying. A seventh item addressing social exclusion from play was also included in the original instrument but excluded from analyses (see below). Six items used a binary response format (Yes = 1 = victimized / No = 2 = not victimized). The seventh item, social exclusion from play, was originally phrased as “Can you join your friends' games without being invited?” (Yes = 1 = no problem / No = 2 = excluded). Because this item assesses passive social withdrawal and self-perceived inclusion capacity rather than active victimization by peers, and because it yielded a negative corrected item-total correlation (r_itc = −0.075) indicating structural divergence from the remaining items, it was excluded from all analyses. Final analyses were therefore conducted on six bullying items. Content validity was established through expert review (two experts in psychological counseling and guidance, one in primary education).

Psychometric properties were examined at the item level using KR-20, the appropriate reliability coefficient for binary-response items. KR-20 for the final six-item scale was 0.582 (*N* = 579). Item difficulty indices (*p*) ranged from 0.085 to 0.339. Corrected item-total correlations (*r* itc) were positive for all six retained items (0.147–0.351). The originally included social exclusion item was excluded prior to analysis because it yielded a negative corrected item-total correlation (*r* itc = −0.075), indicating that it measured a structurally distinct construct (passive social withdrawal) rather than active peer victimization. Excluding this item raised KR-20 from 0.471 to 0.582. Because all analyses were conducted separately for each bullying type—without computing a total score—internal consistency is not the primary reliability index; item difficulty indices and corrected item-total correlations should be interpreted together. Future research is encouraged to use multi-item scales for each bullying subtype.

***Physical Measurements***. Height and weight were measured by the researcher under standardized conditions using a measuring tape and a digital scale. Measurements were taken in the morning while students wore daily clothing without shoes. BMI was calculated as weight (kg) / height^2^ (m^2^).

### BMI classification

BMI values were classified into four categories using WHO (2007) age- and sex-specific growth references: Thinness (<−2 SD), Normal (−2 SD to +1 SD), Overweight (+1 SD to +2 SD), and Obese (> +2 SD). Age- and sex-specific thresholds are presented in Table 2. Due to the insufficient number of students in the Thinness category (*n* = 12), this group was excluded. The Overweight and Obese categories were merged (*n* = 186) for a two-group comparison (Normal BMI vs. Overweight/Obese BMI). In addition, all three groups (Normal, Overweight, and Obese) were compared separately in an exploratory analysis to provide a more complete descriptive picture of the data.

**Table 2:** Age- and sex-specific BMI classification thresholds according to WHO (2007) growth references.

Age	Sex	Thinness	Normal	Overweight	Obese
7	Girl	<12.7	12.7–17.7	17.8–20.8	≥20.9
7	Boy	<12.7	12.7–17.4	17.5–20.5	≥20.6
8	Girl	<12.9	12.9–18.3	18.4–21.8	≥21.9
8	Boy	<12.9	12.9–18.0	18.1–21.5	≥21.6
9	Girl	<13.2	13.2–19.1	19.2–23.1	≥23.2
9	Boy	<13.1	13.1–18.8	18.9–22.7	≥22.8
10	Girl	<13.5	13.5–20.1	20.2–24.5	≥24.6
10	Boy	<13.4	13.4–19.7	19.8–24.2	≥24.3

BMI, Body Mass Index (kg/m^2^). Thresholds are based on (27) growth reference z-scores: Thinness <−2 SD, Normal −2 SD to +1 SD, Overweight +1 SD to +2 SD, Obese > +2 SD.

### Procedure

Parents were informed and provided written informed consent prior to data collection. Data were collected between November 18–24, 2025. The questionnaire was administered during class hours by classroom teachers. Before administration, the researcher informed students about the study, emphasizing confidentiality and voluntary participation. Grades 3 and 4 completed the questionnaire independently using paper and pencil. For Grades 1 and 2, items were read aloud by the researchers to prevent reading comprehension difficulties, and students were guided through each response. To prevent students from influencing one another, Grades 1 and 2 questionnaires were administered individually. Height and weight measurements were conducted on the same day as questionnaire administration.

### Ethical approval

Ethical approval was obtained from Bartin University Social and Humanities Sciences Ethics Committee (Decision No: [2025-SBB-0467], Date: [7.05.2025]). Written consent was obtained from school administration and parents before data collection. Student participation was entirely voluntary.

### Data analysis

Data were analyzed using IBM SPSS Statistics (Version 23) through a three-step analytical strategy. In Step 1, chi-square independence tests examined the relationship between BMI category and each bullying type; effect size was assessed with Cramér's *V*. In Step 2, binary logistic regression tested whether BMI category predicted victimization, reporting odds ratios (OR) and 95% CIs. A second adjusted model controlled for gender and grade level. In Step 3, BMI was treated as a continuous variable and its relationship with each bullying type was examined using Pearson *r* and Spearman ρ correlations. The significance level was set at *p* < 0.05 for all analyses. To address the risk of Type I error from multiple comparisons across six bullying types retained in the final analysis, a permutation test was additionally conducted for the logistic regression analysis (10,000 iterations; seed = 42). This distribution-free approach was selected given the small cyberbullying subgroup size and the binary nature of the outcome variable, conditions under which parametric *p* values may be imprecise. In Step 4, an exploratory subgroup analysis compared all three BMI groups—Normal (*n* = 381), Overweight (*n* = 121), and Obese (*n* = 65)—to provide a more complete descriptive picture of victimization patterns and to assess whether these subgroups carry distinct psychosocial risk profiles. Victimization rates were reported for each group, and Fisher's exact test was used for pairwise comparisons between the Overweight and Obese subgroups. This step was exploratory in nature and was conducted to provide empirical justification for the group-merging decision in Steps 1–3 and to identify any divergent patterns across bullying subtypes.

## Results

The effect of BMI category on peer bullying victimization was examined across four analytical steps. The Thinness BMI category was excluded due to small sample size (*n* = 12). The Normal BMI group comprised *n* = 381 and the Overweight/Obese group comprised *n* = 186, yielding a total analytic sample of *N* = 567. In Step 4, an exploratory analysis compared all three BMI groups — Normal (*n* = 381), Overweight (*n* = 121), and Obese (*n* = 65) — to provide a more complete descriptive picture of bullying victimization patterns and to assess whether the Overweight and Obese subgroups carry distinct risk profiles.

### Step 1: chi-square analysis and descriptive statistics

The relationship between BMI category and exposure to each bullying type was tested using chi-square analysis (see Table 3). Although slightly higher rates were observed in the Overweight/Obese group for physical bullying (33.9% vs. 34.4%), unwanted name-calling (23.4% vs. 25.3%), upsetting pranks (27.6% vs. 29.6%), none of these differences reached statistical significance (all *p* > 0.10). Cyberbullying showed a notably higher rate in the Overweight/Obese group compared to the Normal group (11.8% vs. 6.8%); however, this difference did not reach the significance threshold (χ^2^(1) = 3.419, *p* = 0.064, V = 0.078). The majority of students experienced at least one type of bullying across the six retained items (Normal BMI: 64.8%; Overweight/Obese BMI: 65.6%).

**Table 3:** Distribution of peer bullying types by BMI category, Chi-Square test results, and item psychometric properties (Normal BMI vs. Overweight/Obese BMI).

	Normal BMI			Overweight/Obese BMI								
Bullying type	*N*	*n*	%	*N*	*n*	%	χ^2^	*p*	Sig	V	*p* item	*r* itc
Physical Bullying	381	129	33.9	186	64	34.4	0.001	*0.972*	ns	0.001	0.339	0.278
Taking Belongings	381	87	22.8	186	39	21.0	0.156	*0.693*	ns	0.017	0.219	0.351
Unwanted Nicknames	381	89	23.4	186	47	25.3	0.156	*0.693*	ns	0.017	0.237	0.336
Upsetting Pranks	381	105	27.6	186	55	29.6	0.160	*0.689*	ns	0.017	0.282	0.334
Mockery	381	91	23.9	186	42	22.6	0.057	*0.812*	ns	0.010	0.231	0.242
**Cyberbullying**	381	**26**	**6.8**	186	**22**	**11.8**	**3.419**	***0.064***	†	**0.078**	0.085	0.147
At Least 1 Bullying Type	381	247	64.8	186	122	65.6	0.007	*0.932*	ns	0.004	—	—

*N* = 567. Normal BMI, *n* = 381; Overweight/Obese BMI, *n* = 186. Thinness BMI category (*n* = 12) was excluded. V = Cramér's V (effect size). *p* item = item difficulty index (proportion of students reporting victimization; *N* = 579). *r* itc = corrected item-total correlation (KR-20 = 0.582 for the six retained items, *N* = 579). The social exclusion item was excluded from analysis due to a negative *r* itc = −0.075 (see Method). † *p* = 0.064 (borderline significance). Bold values indicate statistically significant results (*p* < 0.05).

### Step 2: logistic regression analysis

In the unadjusted model (Model 1), overweight/obese students showed a higher likelihood of cyberbullying victimization compared to normal-weight peers [OR = 1.83, 95% CI (1.008, 3.328), *p* = 0.047]. To verify this finding against the risk of Type I error arising from multiple comparisons, a permutation test was conducted (10,000 iterations). The observed OR of 1.83 was exceeded by only 3.2% of random permutations (*p*_perm_ = 0.032), confirming that this association is unlikely to be an artifact of chance. No other bullying type approached significance under permutation testing (all *p*_perm_ > 0.30; see Table 4).

**Table 4:** Logistic regression results for BMI category predicting peer bullying victimization.

Bullying type	*N*	*n*	%	Model 1 (Unadjusted)			Model 2 (Adjusted)			
				OR	95% CI	*p*	OR	95% CI	*p*	Sig	Sig
Physical Bullying	567	193	34.0	1.025	[0.708, 1.483]	0.897	1.009	[0.693, 1.470]	0.964	ns	ns
Taking Belongings	567	126	22.2	0.897	[0.585, 1.373]	0.616	0.904	[0.589, 1.387]	0.643	ns	ns
Unwanted Nicknames	567	136	24.0	1.109	[0.738, 1.667]	0.617	1.086	[0.721, 1.636]	0.694	ns	ns
Upsetting Pranks	567	160	28.2	1.104	[0.750, 1.625]	0.618	1.198	[0.807, 1.779]	0.369	ns	ns
Mockery	567	133	23.5	0.929	[0.613, 1.410]	0.731	0.933	[0.613, 1.419]	0.746	ns	ns
**Cyberbullying**	567	**48**	**8.5**	**1.832**	**[1.008, 3.328]**	**0.047**	**1.768**	**[0.969, 3.227]**	**0.064**	^*^	†
At Least 1 Bullying Type	567	488	65.1	1.006	[0.726, 2.055]	0.981	1.049	[0.763, 2.180]	0.848	ns	ns

*N* = 567. Reference category: Normal BMI. Model 2 included gender (0 = girl, 1 = boy) and grade level (1-4) as covariates. OR = Odds Ratio; CI = Confidence Interval. ^*^p < 0.05. †*p* = 0.064 (borderline significance). Bold values indicate statistically significant results (*p* < 0.05).

In the adjusted model (Model 2), controlling for gender and grade level, the odds ratio for cyberbullying declined to borderline significance [OR = 1.77, 95% CI (0.969, 3.227), *p* = 0.064], suggesting that part of the crude BMI effect on cyberbullying operates through demographic variables.

### Step 3: correlation analysis

Treating BMI as a continuous variable, only cyberbullying showed a significant relationship with BMI scores (ρ = 0.092, *p* = 0.027; see Table 5). Although the effect size was small, the direction was positive and consistent with the permutation-validated logistic regression finding. Pearson correlation yielded a similar pattern (*r* = 0.087, *p* = 0.037). Correlation coefficients for the remaining six bullying types were near zero (all *p* > 0.20).

**Table 5:** Pearson and spearman correlation coefficients between BMI and peer bullying types.

Bullying Type	*N*	Pearson *r*	*p*	Spearman ρ	*p*	Sig	Interpretation
Physical bullying	579	*−0.027*	*0.524*	*−0.026*	*0.529*	ns	—
Taking belongings	579	*−0.030*	*0.469*	*−0.020*	*0.633*	ns	—
Unwanted nicknames	579	*0.018*	*0.668*	*0.008*	*0.855*	ns	—
Upsetting pranks	579	*−0.060*	*0.149*	*−0.053*	*0.206*	ns	—
Mockery	579	*0.010*	*0.802*	*0.044*	*0.293*	ns	—
**Cyberbullying**	579	***0.087***	***0.037***	***0.092***	***0.027***	^*^	**Significant positive**

*N* = 579 (all valid BMI values included). BMI used as a continuous numeric variable. ^*^p < 0.05. Bold values indicate statistically significant results (*p* < 0.05).

### Step 4: exploratory subgroup comparison (overweight vs. obese)

Taken together, BMI category did not predict general peer bullying victimization in this sample. Cyberbullying was the only consistent exception across all three methods. Critically, permutation testing confirmed that the unadjusted OR of 1.83 was unlikely to be a chance finding (*p*_perm_ = 0.032), providing additional robustness beyond the nominal *p* value. The decline to borderline significance after demographic adjustment (OR = 1.77, *p* = 0.064) suggests that gender and grade level partly mediate this relationship rather than confounding it entirely.

Exploratory subgroup analyses separating the Overweight (*n* = 121) and Obese (*n* = 65) groups revealed divergent patterns across bullying types (see Table 6). Cyberbullying showed a monotonically increasing pattern across BMI categories (Normal: 6.8%, Overweight: 10.7%, Obese: 13.8%), suggesting a dose-response relationship and supporting the rationale for merging these groups in the primary analyses. In contrast, upsetting pranks showed a V-shaped pattern (Normal: 27.6%, Overweight: 37.2%, Obese: 15.4%), with Overweight and Obese subgroups differing significantly (Fisher's exact *p* = 0.002). This divergence indicates that these two subgroups may carry distinct psychosocial risk profiles for certain bullying subtypes. For all other bullying types, the Overweight and Obese groups showed similar rates with no statistically significant between-subgroup differences (all Fisher's exact *p* values ≥ 0.36), providing empirical justification for their pooling in the primary analyses. These exploratory findings underscore the need for larger samples that permit robust separate subgroup analyses.

**Table 6:** Exploratory subgroup comparison: bullying victimization rates across normal, overweight, and obese BMI categories.

Bullying type	Normal (*n* = 381)	Overweight (*n* = 121)	Obese (*n* = 65)	OW vs. OB *p* (Fisher)	Pattern
	*n* (%)	*n* (%)	*n* (%)		
Physical bullying	129 (33.9)	43 (35.5)	21 (32.3)	0.747	Irregular
Unauthorized taking	87 (22.8)	26 (21.5)	13 (20.0)	0.853	↓ Decreasing
Unwanted name-calling	89 (23.4)	31 (25.6)	16 (24.6)	1.000	Irregular
Upsetting pranks	105 (27.6)	**45 (37.2)**	**10 (15.4)**	**0.002**	***V-shaped** **▴***
Mockery	91 (23.9)	30 (24.8)	12 (18.5)	0.363	Irregular
Cyberbullying	26 (6.8)	13 (10.7)	9 (13.8)	0.635	*↑ Monotonic*
At least 1 type	247 (64.8)	80 (66.1)	42 (64.6)	1.000	Irregular

OW, Overweight; OB, Obese. Fisher's exact test used for Overweight vs. Obese comparisons. † Thinness group (*n* = 12) excluded from all analyses. ▴ V-shaped pattern: Overweight > Normal > Obese; Overweight vs. Obese difference statistically significant (*p* = 0.002). Bold values indicate statistically significant results (*p* < 0.05).

## Discussion

This study examined the relationship between BMI and types of peer bullying in primary school students, yielding findings that both align with and illustrate the complexity of current literature. BMI category did not predict general peer bullying victimization. Cyberbullying was the only consistent exception across methods; however, this association also declined to borderline significance when demographic variables were controlled.

The association between cyberbullying and BMI supports the view that obesity is not merely a physical condition but a psychosocial phenomenon shaped by digital and social environments. Childhood obesity arises from the complex interplay of biological, environmental, and maternal factors (1). Elevated BMI, rising cortisol levels, and systemic inflammation create a maladaptive cycle by triggering chronic stress and mental health problems (17, 18). The relationship between bullying victimization and obesity development in children aged 11–14 has been linked to biological responses and unhealthy coping mechanisms such as emotional eating (19).

Social stressors such as stigmatization and peer bullying lead overweight children to withdraw from the social world and turn toward virtual environments, making digital spaces a form of escape or coping mechanism (19). As described by Farrell et al. (18), this isolation is not simply an inward turning but a state of “not fitting in,” arising from the physical world's failure to accommodate the obese individual. As the child increasingly retreats to digital environments, the risk of online bullying grows. Adolescents who experience peer bullying become more prone to internet addiction due to increasing loneliness, and overweight children in digital environments are more frequently targeted by cyberbullying (8). These findings are consistent with our result that children in the Overweight/Obese BMI group were more likely to experience cyberbullying (OR = 1.83, Model 1; *p*_perm_ = 0.032).

Across both BMI groups, physical bullying was the most prevalent type (Normal: 33.9%, Overweight/Obese: 34.4%), with virtually no between-group difference. This is consistent with literature showing that physical bullying at the primary level is driven more by gender and behavioral dynamics than by body weight (5, 6). Boys tend to assert dominance through physical bullying when rejected by peers, while girls more often resolve conflicts cooperatively (20). The tendency to model aggressive behaviors from preschool age onward (21), combined with increasing peer rejection of overweight children as grade level rises (13), makes breaking this cycle increasingly difficult.

Bullying victimization experienced by children living with obesity represents deep psychological damage operating through self-esteem. Lovan et al. (22) found that victimization increases anxiety and depression, with self-esteem serving as a central mediating variable—particularly in girls. The cyberbullying risk identified here could, over time, erode children's self-esteem and draw them into intense psychological suffering without a clinical diagnosis (17). Childhood maltreatment can increase both victimization and perpetration by approximately 3-fold (23), and in environments where aggression is normalized, victims may eventually shift to the perpetrator role (15, 24).

It is well established that children during primary school years are profoundly influenced by their social environment (25). The value a child assigns to themselves may sometimes reflect the level at which they are perceived by peers or teachers (26). Children who are bullied by peers can become trapped in a vicious cycle. When verbal, physical, or cyberbullying by peers is ignored by teachers—or reinforced through joking remarks—a period of stigmatization begins for the student. Even when such labeling is well-intentioned (e.g., assigning affectionate nicknames related to body weight, treating excess weight as endearing, or making gestures that suggest an obese child is somehow more likable because of their size), teacher behavior is known to directly trigger peer bullying (14). It is therefore critical that educators avoid stigmatizing language when addressing students about obesity. A widespread belief across many societies holds that obesity is an illness and that one must be especially careful not to hurt the feelings of individuals with obesity. The exploratory findings from Step 4 of this study provide compelling evidence consistent with this view.

### Limitations and future directions

Several limitations should be considered. The decline of BMI's effect on cyberbullying to borderline significance when demographic variables are controlled (OR = 1.77, *p* = 0.064) suggests that this relationship partly overlaps with gender and grade level. A permutation test (10,000 iterations) was applied to the logistic regression to address multiple comparison concerns without relying on parametric distributional assumptions; cyberbullying remained significant under this test (*p*_perm_ = 0.032). The precision of the primary estimate is limited: the 95% CI borders unity [OR = 1.83, 95% CI (1.008, 3.328)], reflecting the small number of cyberbullying events (*n* = 48). *Post-hoc* power analysis indicated that the study achieved only 52% power to detect an effect of this magnitude at α = 0.05; achieving 80% power would require approximately 1,294 participants. Larger-sample studies are therefore needed to improve precision, narrow the confidence interval, and rule out Type II error for the remaining bullying types. The sample was drawn from a single public school in Bartin, which limits external validity. The binary response format (Yes/No) has limited capacity to capture nuanced information about bullying frequency and severity. The social exclusion item was excluded from analyses due to a negative corrected item-total correlation (r_itc = −0.075), which raised KR-20 from 0.471 to 0.582; however, single-item indicators remain a limitation and future research should employ multi-item scales for each bullying subtype. Longitudinal designs are needed to examine the mechanisms mediating the BMI–cyberbullying relationship (social isolation, self-esteem, internet use).

## Data Availability

The original contributions presented in the study are included in the article/supplementary material, further inquiries can be directed to the corresponding author.
